# Creative Persuasion: A Study on Adversarial Behaviors and Strategies in Phishing Attacks

**DOI:** 10.3389/fpsyg.2018.00135

**Published:** 2018-02-21

**Authors:** Prashanth Rajivan, Cleotilde Gonzalez

**Affiliations:** Dynamic Decision Making Laboratory, Social and Decision Sciences, Carnegie Mellon University, Pittsburgh, PA, United States

**Keywords:** phishing, adversarial behavior, strategy, deception, creativity, persuasion, simulation

## Abstract

Success of phishing attacks depend on effective exploitation of human weaknesses. This research explores a largely ignored, but crucial aspect of phishing: the adversarial behavior. We aim at understanding human behaviors and strategies that adversaries use, and how these may determine the end-user response to phishing emails. We accomplish this through a novel experiment paradigm involving two phases. In the adversarial phase, 105 participants played the role of a phishing adversary who were incentivized to produce multiple phishing emails that would evade detection and persuade end-users to respond. In the end-user phase, 340 participants performed an email management task, where they examined and classified phishing emails generated by participants in phase-one along with benign emails. Participants in the adversary role, self-reported the strategies they employed in each email they created, and responded to a test of individual creativity. Data from both phases of the study was combined and analyzed, to measure the effect of adversarial behaviors on end-user response to phishing emails. We found that participants who persistently used specific attack strategies (e.g., sending notifications, use of authoritative tone, or expressing shared interest) in all their attempts were overall more successful, compared to others who explored different strategies in each attempt. We also found that strategies largely determined whether an end-user was more likely to respond to an email immediately, or delete it. Individual creativity was not a reliable predictor of adversarial performance, but it was a predictor of an adversary's ability to evade detection. In summary, the phishing example provided initially, the strategies used, and the participants' persistence with some of the strategies led to higher performance in persuading end-users to respond to phishing emails. These insights may be used to inform tools and training procedures to detect phishing strategies in emails.

## 1. Introduction

Many successful cyber attacks begin with *social engineering*—use of psychological manipulations to trick people into disclosing sensitive information or inappropriately granting access to a secure system (Anderson, 2010). Social engineering is perhaps, the most convenient method to breach a secure network which is otherwise difficult to breach through technological means (Forest, 2017). Phishing is a common kind of social engineering attack, where criminals impersonate a trustworthy third party to persuade people to visit fraudulent web sites or download malicious attachments; actions which compromise people's own security and possibly an organization's security.

There has been a resurgence in phishing attacks in recent years. For example, a 250% increase in new attacks was observed in 2016 alone (APWG, 2016), and an average of 1.4 million unique phishing websites are being created each month (Webroot, 2017). Phishing attacks have also become more effective, more sophisticated, and difficult to detect with existing anti-phishing tools. New phishing techniques are scaling up from traditional monetary scams to targeted attacks. For example, *spear-phishing* is a targeted type of phishing that relies on context-specific, carefully crafted emails directed at specific organizations or individuals (Schuetz et al., 2016). Effective spear-phishing attacks go beyond the usual tricks of visual deception, and require that end-users pay attention to the plausibility of the message, making it more difficult to detect.

Several techniques are necessary to combat phishing attacks, including end-user training and automated anti-phishing tools. However, these methods are not fully effective. Anti-phishing training procedures are often less effective, because people generally perceive security as a secondary, low-priority task (Krol et al., 2016; Schuetz et al., 2016). Long-term training is necessary to reasonably reduce human susceptibility to phishing emails (Wombat, 2016; Ben-Asher et al., in preparation). Moreover, existing training procedures may not be effective against sophisticated attacks because it mostly teaches people basic heuristics (e.g., suspicious from-address, typographical errors, lock icons), and encourage unrealistic protective actions such as “do not click on links in emails” (Downs et al., 2006; Hong, 2012). Automated solutions are preferred, but being able to automatically flag the more sophisticated phishing attacks is becoming a tremendous challenge because the current tools rely primarily on technical characteristics of emails that can be easily perturbed by sophisticated adversaries to evade detection (Ma et al., 2009; Felegyhazi et al., 2010; Huang et al., 2011; Shekokar et al., 2015; Liang et al., 2016; Schuetz et al., 2016). For example, adversaries launch several new phishing websites when existing ones are blacklisted. Therefore, we need to advance phishing detection, both automated tools and human training, to look beyond simple heuristics and transient email characteristics.

Like many challenges in cybersecurity, phishing attacks need to be addressed by building solutions that are informed by the psychology of human behavior (Gonzalez et al., 2014). However, much of the past psychological studies of phishing behavior have concentrated on the end-user. These studies have discovered a variety of factors that influence end-user reaction to phishing emails. For example, phishing emails are often superficially processed, leading to decision making that is based on deceptive visual cues, and strategies such as urgency and trust (Dhamija et al., 2006; Downs et al., 2006; Vishwanath et al., 2011; Hong, 2012; Lastdrager, 2014). Limited knowledge, lack of attention to security cues, and habituation are among numerous other potential contributors for end-user susceptibility to phishing attacks (Dhamija et al., 2006; Kelley et al., 2016; Vishwanath et al., 2016).

To combat sophisticated phishing attacks, we need to look beyond end-user behaviors. Psychological research on human adversarial behavior is necessary to uncover factors that determine how deception and phishing strategies originally manifest in phishing emails (Abbasi et al., 2016). Currently, there is a severe lack of work on the psychology of criminal behaviors in cybersecurity. This research contributes to an understanding of adversarial behaviors and traits in phishing attacks including: the role of incentives in phishing attacks; the role of attacker's creativity as a predictor of success in phishing attacks; and the effect of adversarial strategies on attack success.

### 1.1. Research questions

Research question 1: What role do incentives play in determining phishing effort and success? It is clear that adversaries are motivated by various incentives; they may be financial, informational, or political. Therefore, payoffs gained from attacks play an important role in moderating adversarial behaviors. However, higher incentives may not be associated with more lying or deception (Mazar et al., 2008; Fischbacher and Föllmi-Heusi, 2013). Like in physical crime, delay in payoffs could affect dishonest behaviors. Individuals indulging in dishonest behaviors have been shown to prefer smaller yet immediate rewards over larger delayed ones (Kirby and Maraković, 1995; Frederick et al., 2002; Wu et al., 2017). We currently do not understand what effects a delay in the rewards has on the effort adversaries exert in their attacks. Therefore, this research explores whether early rewards motivate attackers to put more effort in the subsequent attack attempt or whether delayed rewards would keep attackers interested in exerting more effort to achieve a high reward.

Research question 2: Does individual creativity predict success in phishing attacks? Creativity is traditionally seen as a “good” trait because it represents the ability to generate novel ideas, and creative solutions that can enable individuals to adapt effectively to new problems and challenges (Mumford and Gustafson, 1988; Flach, 1990). However, a growing body of research shows that higher creative ability could also enable individuals to engage in unethical or dishonest behaviors (Gino and Ariely, 2012; Beaussart et al., 2013; Cropley and Cropley, 2013). Dishonest and unethical behaviors that have been associated with higher creativity include: lying to supervisor about work progress, stealing from work, and falsely reporting higher performance on lab-based, experimental tasks to earn more money. This research suggests that creative individuals have higher ability to self-justify their dishonest actions (Gino et al., 2013; Shalvi and De Dreu, 2014). Similarly, hackers are also characterized as creative and unconventional individuals with a propensity for lying and deceit driven by self-justified motivations (Nikitina, 2012; Steinmetz, 2015). However, there has been no empirical research on the effect of creativity in determining the success of cyberattacks. Therefore, in this study, we explore the role of individual creativity in phishing attacks, specifically to determine whether creative ability relates to higher success with phishing attacks.

Research question 3: What, and how strategies are implemented in successful phishing emails? A primary aspect of a phishing email that achieve social engineering is deception through persuasion strategies, such as a sense of urgency, or authoritativeness (Ferreira and Lenzini, 2015; Ferreira et al., 2015; Harris and Yates, 2015; Zielinska et al., 2016). Some of these adversarial strategies may be more likely than others to elicit immediate response from end-users (Wombat, 2016). For example, emails seemingly real, and relevant to work and personal life, such as communications about work documents, tax documents, benefits documents, usually have the highest success rate (Wright et al., 2014). Also, email that appears to be from an acquaintance, are also likely to produce a response (Parsons et al., 2015). Therefore, in this work, we study the adversarial side of phishing strategies to explore how strategies are employed by attackers in phishing emails; how attackers learn from different attempts; whether exploring different strategies lead to higher attack performance and whether certain kinds of strategies lead to higher attack success.

In what follows, we introduce a research paradigm, discuss materials and methods of an experimental study, and present results that provide answers to these research questions on adversarial behaviors in phishing.

## 2. Materials and methods

**Figure 1:**
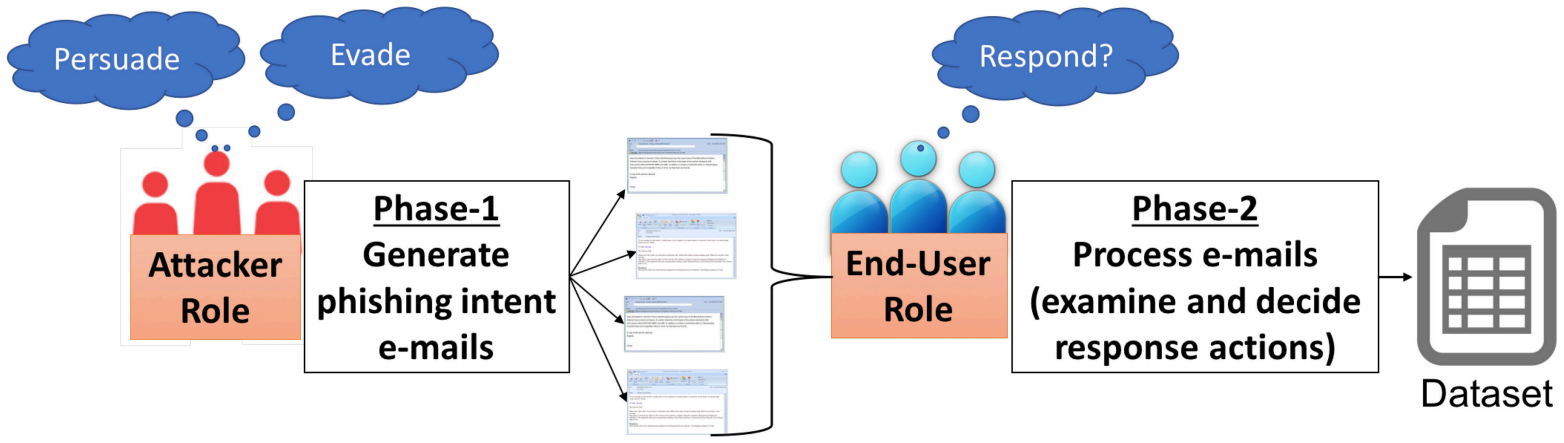
Overview of the paradigm designed to study adversarial behaviors and strategies in phishing.

As represented in the Figure 1, we conducted a two-part study on adversarial behaviors and strategies that predict phishing attack performance. Phase-1 of the study involved human participants playing the role of a phishing attacker, creating and launching multiple phishing emails. Phase-2 of the study involved participants playing the role of an end-user, making repeated decisions on multiple emails which include phishing emails created by participants in Phase-1 along with benign, non-phishing (ham) emails. Two studies were conducted independently, one after the other; Phase-2 was conducted following the data collected from Phase-1.

### 2.1. Study-phase 1: phishing attacker

#### 2.1.1. The phishing attacker console

To study the behaviors of a phishing attacker, we designed a simulation environment and an experimental paradigm. In building this simulation, we made few assumptions regarding the tasks and goals of adversaries launching phishing emails. We assumed that a phishing adversary (i.e., “phisher”) would need some infrastructure to design, develop, and launch phishing attacks; and that the phisher pursued the goal of maximizing rewards (financial or otherwise). To maximize rewards, phishers would perform the following tasks:

- Write and launch multiple phishing email attacks.
- Write emails that are able to evade detection technology (e.g., spamassassin).
- Write strategic emails that would persuade multiple human recipients to respond.

Participants acting as phishers were tasked with launching a series of phishing emails (each attempt called a “trial”), targeting the evasion of fictional, detection technologies, and aiming at persuading end-users to engage in risky online behaviors. Using a simulation interface, they were asked to write multiple phishing emails and launch each of the phishing emails they crafted. The simulation provided feedback regarding the success of an attack in the form of a reward (i.e., points earned/lost), as well as the accumulated rewards across trials. Participants were rewarded for evading detection, and persuading end-users to respond. The goal in the simulation was to maximize overall individual rewards.

To evade detection participants were encouraged to edit and modify the content of their phishing emails, so that they would explore the effectiveness in each attempt. The simulation provides an initial template which participants modify in their first attempt. The simulation also allows participants to edit the email launched in the previous attempt or to write a new phishing email from scratch. Participants are encouraged to make changes to the emails before submitting an email for evaluation, according to the design of rewards. The reward for evading detection is calculated in direct proportion to the number of edits made in each trial.

The number of edits are calculated using a standard distance function between a pair of character strings called the Levenshtein edit distance. This function calculates the number of characters that need to be inserted, deleted and substituted to derive one string from another. Edit distance functions are usually used in text and speech processing. For example, it is used to detect plagiarism in a text-based document, and evaluate the actual human effort (Zini et al., 2006; Su et al., 2008). Levenshtein distance between two strings A,B of length i and j respectively is calculated using the recursive function shown in Algorithm 1.

**Algorithm 1:** Computing Levenshtein edit distance

$$\begin{array}{l} D(i,j)=\min\{D(i-1,j-1)+\gamma (A\langle i\rangle \to B\langle j\rangle ),\\ \;D(i-1,j)+\gamma (A\langle i\rangle \to \Lambda ),\\ \;D(i,j-1)+\gamma (\Lambda \to B\langle j\rangle )\} \end{array}$$

In the simulation, we used this algorithm to calculate the Levenshtein edit distance between email text submitted in each trial with the email text produced in the previous trial; effectively measuring the amount of changes made in each trial. In each trial, participants could earn a maximum of 200 points and a minimum of 0 points as rewards for evasion.

**Figure 2:**
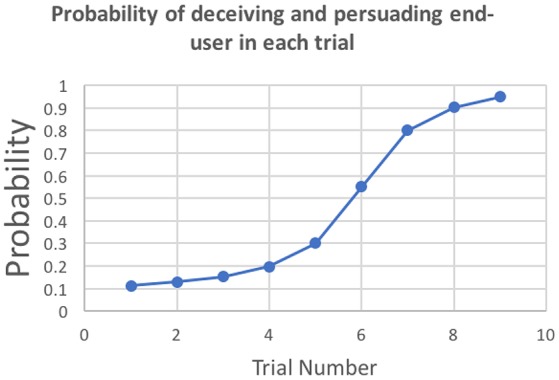
Probability of rewards for persuading end-users to respond in each trial.

The reward for persuading the end-user to respond was the result of a probabilistic function. We designed this function such as the likelihood of winning the reward increased with each trial as shown in Figure 2. This was done to keep the participant's interest and to reflect the naturalistic probabilistic nature of the success of a phishing attack. The reward for a successful attack was a one-time, high-value payoff of 2,000 points. Participants are eligible for earning this reward only if they had done a minimum of 50 edits in the submitted email. This threshold of 50 edits was chosen based on the pilot experiments. If the edits to the email were above 50, then a random draw from the probability associated to the trial number determined whether the participant obtained the 2,000 points. We will refer to the trial number in which they received the high-value reward as *High-Value Reward Trial*.

The overall reward function in each trial is as follows: *Reward*_*t*_ = *Gain*_*dt*_ + *Gain*_*et*_ − *Cost*_*t*_ where t = Trial, *Cost*_*t*_ is the Cost of launching the attack in the trial t, *Gain*_*dt*_ is the rewards gained for persuading end-user in trial t, *Gain*_*et*_ is the rewards gained for evading detection in trial t; *Reward*_*t*_ is the net-rewards received in trial t. The net reward from a trial was added to points accumulated by the participant up to trial *t* − 1. Hence, *Capital*_*t*_ = *Capital*_*t*_ − 1 + *Reward*_*t*_ where *Capital*_*t*_ − 1 = accumulated points the participant has at the beginning of trial t and *Capital*_*t*_ = points the participant carries on to the next trial that included the rewards received in the trial t. At the end of the trial, participants received feedback of the total reward and cost of the attack launched (which is a constant 200 points) and the total gains received from launching the attack in that trial. In addition, the simulation provides the cumulative reward.

#### 2.1.2. Pilot experiment

A pilot experiment was conducted in a laboratory with 10 participants, to evaluate the simulation software, experiment design and procedure. Participants for the pilot experiment were university students, recruited from the university participant pool and through online advertisements. We paid $12 for their time and effort. Observations and analyses of the laboratory pilot experiment suggested reduction in the number of trials from 10 to 8 to ensure adequate workload for participants; increasing of the threshold for eligibility for high-value rewards from 0 to 50 character edits per trial to ensure participants are rewarded fairly according to their effort in the experiment; and inclusion of a standardized instrument to measure individual personality traits (Jones and Paulhus, 2014). The choice of 50 edits as a threshold was a subjective choice, based on manual inspection of the emails from pilot experiment which indicated that at least 50 edits was necessary to add a new argument or rhetoric to an existing phishing email. These design and procedural changes were included in the full experiment, described next.

#### 2.1.3. Study design and procedures

Phase-1 of the full-study involved participants from Amazon MTurk (Buhrmester et al., 2011). Participants were limited to only those residing in the US. 105 MTurk participants were given instructions, including the purpose of the study and the role the participants would play. Participants received instructions on the use of phishing emails to steal personal information from people and the instructions necessary for performing the task using the simulation. Phishers were instructed to use their intuition to decide what strategy would be most effective in persuading the end-user to click a link in the email and provide personal information. Phishers were told that attackers usually employed strategies that exploited weaknesses in human emotions (e.g., greed, curiosity, obedience to authority, urgency), pretended to be friends or acquaintances, offered help, informed end-users of a failure or problem, or set deadlines for eliciting immediate response.

All experiment protocols were approved by the university institutional review board (IRB). Each participant conducted eight trials of phishing attacks. Participants started the study with 2,000 points. In each trial, they paid the constant cost for launching the attack, and they received feedback after launching each attack, as described above. At the end of 8 trials, participants could accumulate between 0 and 4,000 points. The final cumulative points directly affected the participant's bonus payment at a rate of $1 for 1,000 points. Their cumulative earnings were added to a $1 base payment. The number of trials and reward structure used in this study were determined after multiple rounds of pilot testing.

In the experiment, participants were first presented with the IRB approved informed consent form. They were told their participation was voluntary and were allowed to participate only if they self-reported they were 18 years or older, consented (by clicking “yes” on the online interface) that they read and understood the information presented in the consent form and wished (voluntarily) to participate in the experiment. After providing consent, they entered basic demographic information such as age, gender and whether they were native English speakers. Participants were also asked to rate their English writing proficiency using a simple 5-point scale which ranged from Beginner (who can write few words and partial sentences) to intermediate to Very-Advanced (who can write with perfect grammar, and always convey thoughts clearly). We did not collect any additional demographic information such as security expertise or educational-level because they were not central to the goals of this current experiment.

Next, participants were presented with the Guilford's Alternative Uses Test, an established measure individual's divergent thinking ability (Runco and Acar, 2012). We administered this test in our study using the usual procedure where participants were asked to list as many possible uses for a “plank of wood” under 2 min (other common household items are also used in other experiments). User responses for this test are typically scored for performance along four dimensions of creativity/divergent thinking: Originality, Fluency, Flexibility and Elaboration.

After completing the 2-min divergent thinking test, participants were given a brief introduction about phishing attacks in general. They were provided detailed instructions about the task, the reward structure and the task goals. All the training material was presented in a concise, jargon-less manner using visual aids and real-world examples, such that participants with little to none experience with phishing can quickly comprehend it. They were administered an short quiz on the training material presented, to primarily help them reflect the training received and only when they scored a perfect score on this brief quiz, they were allowed to proceed with the experiment. Finally, participants were presented with few practice trials of the simulated phishing tasks they were about to perform.

After practice trials, each participant was randomly assigned a real-world phishing email selected from a phishing corpus shared by Nazario (2016) and from other online sources (e.g., news articles) and security forums. The randomly assigned phishing email in the first trial served as an example or template to help participants develop other phishing emails in their subsequent trials. We used ten emails as *phishing examples*, each representing a variant of phishing attacks commonly encountered, under different topics such as attacks that target consumer accounts (e.g., email communicating about a locked account); attacks that target corporate accounts (e.g., emails requesting to verify work information); tax refund scams; fake job requests scams; fake order placements; fake rewards; and loan scams. Different examples were assigned to inspire participants to create phishing emails under different topics and contexts; and to create a heterogeneous phishing email dataset for evaluation. The ten phishing examples used in the experiment did not differ significantly in their structure or word count (*min* = 95, *mean* = 111, *max* = 137, *SD* = 14).

Each participant performed 8 trials of phishing attacks. In each trial, participants crafted and submitted phishing emails and received feedback on its success as described above. Additionally, in each trial, after submitting the email, they selected all applicable persuasion strategies they had employed in that specific attempt. They were presented with 14 possible options to choose from, presented in random order in each trial (see Table 1). The strategies used in this study were not an exhaustive list, but a subset of known strategies, often qualitatively observed to be associated with phishing emails (Harris and Yates, 2015; Phishme, 2016).

**Table 1:** List of 14 strategies presented to participant in each trial and the keyword reference.

**Strategy**	**Keyword**
Present deadlines	*Deadline*
Use positive emotion (e.g., surprise, excitement)	*Positive*
Use negative emotion (e.g., fer, panic, threat)	*Negative*
Pretend to be a government/workplace authority	*Authority*
Pretend to be a friend/colleague/acquaintance/relative	*Friend*
Pretend to have shared interest (work or activity)	*Interest*
Inform problem/failure/loss	*Failure*
Offer deal/lottery/reward	*Deal*
Present reminder/update/notification	*Notification*
Sell illegal material (e.g., pornography, drugs)	*Illegal*
Present opportunity (job, product or service)	*Opportunity*
Request help	*RHelp*
Offer help	*OHelp*
Other	*Other*

Finally, after the last trial, participants were presented with a standardized personality instrument called the Dark Triad that measures dark personality traits of individuals such as machiavellianism, narcissism, and psychopathy (Jones and Paulhus, 2014). Results related to this individual differences measure are reported in a separate manuscript (Curtis et al., in preparation).

### 2.2. Study phase-2: end-user

Phase-2 of the study was conducted independently, following the completion of phase-1 described earlier. 340 participants were recruited from Amazon's Mechanical Turk, to fill the role of the end-user. Sample size of 340 was specifically chosen to satisfy the requirement of five end-user response for each phishing email produced from Phase-1. All participants received a base payment of 1.50 for participation and they were not incentivized for performance.

In phase-2, participants played the role of an end-user. They were instructed that the goal of the study was to understand how people manage their e-mails. They were presented with 20 e-mails; 10 of the e-mails were benign in nature (ham emails), while the other 10 were phishing e-mails, created and edited by participants in phase-1. The main task in phase-2 was to examine each email and choose a response action, with the aim of assisting a fictional office manager, named “Sally,” to process her Inbox. This is a standard approach used for conducting phishing studies with end-users (see Sheng et al., 2007; Parsons et al., 2015). When participants are made aware that the experiment is about differentiating phishing emails, they are more likely to be produce more false alerts (Parsons et al., 2015). For each email, they were asked to respond with one of five possible actions: Respond Immediately (1); Flag the email for follow up (2); leave the email in the inbox (3); Delete the email (4); Delete the email and block the sender (5).

#### 2.2.1. Phishing email distribution

**Figure 3:**
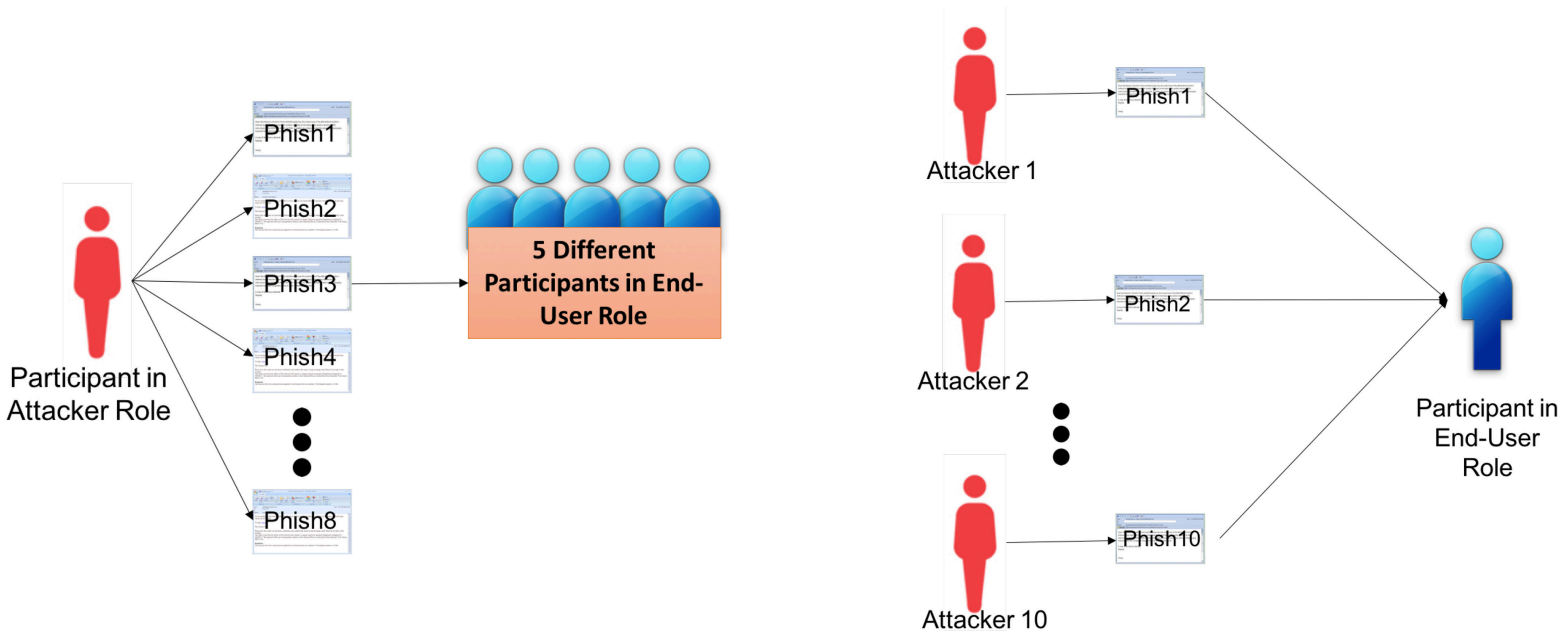
Visual representation of distribution of phishing Emails to participants in Phase-2.

We employed a custom algorithm for random assignment of phishing emails generated from phase-1 to participants in end-user role in phase-2. Such a randomization algorithm was used to ensure that each end-user participant received 10 unique phishing emails from participants in phase-1, potentially containing different strategies and rhetoric. The algorithm was a conditional random assignment of phishing emails to each participant in phase-2 such that:

- Each eligible e-mail (50 edits or more) from phase-1 was distributed to five different participants in phase-2. For example, see Figure 3 where the email identified as “Phish3” from one attacker is shown to be distributed to five different end-users.
- The 10 phishing emails received by each end-user participant in phase-2 were created by 10 different participants (attacker) in phase-1. In other words, only one email from the same attacker participant was presented to an end-user. For example, see Figure 3 the end-user is shown to receive 10 phishing emails, Phish1 to Phish10, from 10 different attackers.
- Furthermore, the 10 phishing emails received by each end-user were created by different attackers who started with different phishing examples in the first trial.

Such a conditional random assignment ensured that participants in the end-user role responded to a variety of phishing emails from different participant sources and therefore, less likely to introduce variance from learning effects and other confounds.

## 3. Results

In phase-1 of the study, we received responses from almost equal number of men (52%) and women (48%) between the ages of 18 and 75. Specifically, we received 52.9% of responses from participants in the age group of 26–35, 23.5% of responses from the age group of 18–25, 15.6% of responses from the age group of 36–45 and remaining from participants in the age groups of 46–55. We received one response each from participants in the age group of 56–65 and 66–75, respectively. All the participants were native English speakers (self-reported). 77.4% of participants rated their English writing proficiency as “Very-Advanced” (Can write with perfect grammar, and always convey thoughts clearly), 17.8% of participants rated their proficiency as “Advanced” (Can write well using appropriate grammar but may still make mistakes and fail to convey thoughts occasionally), and 4.8% of participants self-reported to be at an intermediate level (Can write reasonably well and can use basic tenses but have problems with more complex grammar and vocabulary). In phase-2 of the study, with the end-user, we did not collect any demographic information because it was not relevant to the goals of the experiment.

### 3.1. Analytic approach

**Figure 4:**
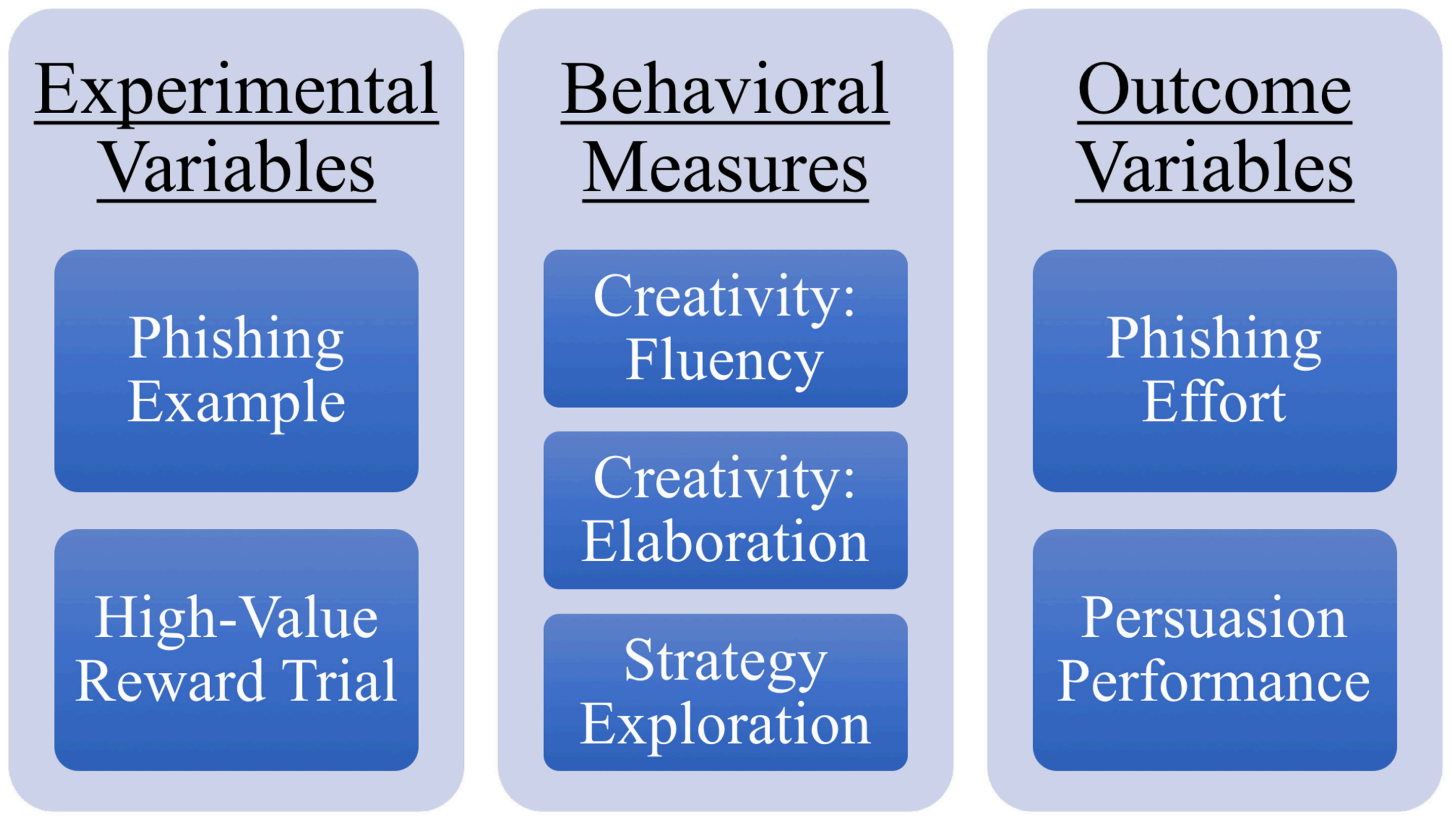
Summary of all the measures used in the analyses. The two experiment variables and three behavioral measures are used to compare and predict the two outcome variables.

Data collected from Phase-1 and Phase-2 of the study was combined to create a synthetic phishing dataset that was labeled; classified based on end-user response to each eligible email; and contains multiple attributes that characterizes attacker behaviors and strategies associated with each email. For participants in the attacker role, we analyzed the effects of 2 experimental variables: the type of example email that was provided to the participants in the first trial (referred as *Phishing Example*) and the trial number in which they received the high-value reward (referred as *High-Value Reward Trial*). We had 2 measures of creativity (fluency and elaboration), and strategies they selected after each email. We also developed a measure to quantify the exploration of different strategies in each trial. We describe this measure in the section titled “Strategy Exploration”. We also had 2 outcome measures: phishing effort (number of changes made in the emails) and persuasion performance (whether the email was effective in deceiving the end-user or not), which are described in the section titled “Phishing Effort and Persuasion Performance.” Figure 4 gives an overview of these factors.

We compared and predicted the performance of participants in the attacker role in terms of both phishing effort and persuasion performance. Specifically, in the section titled “Phishing Effort” we present results from the comparison of phishing effort of participants who received different phishing examples and the high-value reward in different trials. We also present results from a multiple regression analysis that was used to predict phishing effort in terms of individual creativity and strategy exploration. In the section titled “Phishing Performance” we present results from a similar set of analyses using the same set of independent variables but using Persuasion performance as the dependent variable. In the section “Association Between Strategies,” we transition to the analysis of strategies used in the phishing emails to measure the relationship between different strategies; and the relationship between individual strategies and end-user response to them.

### 3.2. Strategy exploration

**Figure 5:**
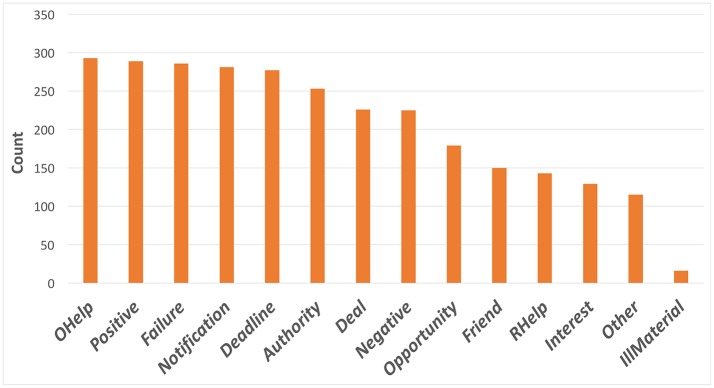
Comparison of total number of times each strategy was used across all emails.

We analyzed the strategies that participants reported as having been used in each trial. In the majority of the instances, participants reported they used more than 1 strategy in each email. Figure 5 is the distribution of the reported persuasion strategies (refer Table 1 for strategy description for each keyword in the graph). The “Sell illegal material” was the least commonly used persuasion strategy, while strategies such as “Inform failure,” “Offer help,” “Present reminder/notification,” and “Use positive emotion” were the most common. This analysis is informative as it indicates the frequency of use of different strategies on average. However these analyses do not indicate how effective those strategies were, and also the individual variability in the use of the strategies. We analyze these issues next.

In the data we observed large variability in the use of strategies across participants. For example, there were participants who used only positive and opportunistic strategies in all their trials whereas other participants self-reported to have to used eight different strategies across the 8 phishing attempts. We quantified this exploratory behavior as follows.

Let *S*_*ij*_ represent the total number of times a strategy *i* was used by participant *j* across all 8 trials. Let *V*_*j*_ represent a vector of *S*_*ij*_ for participant *j* where *V*_*j*_ is a vector of length 14 that contains the total number of times each of the 14 strategies was used by participant *j*. Therefore, the measure of exploration for participant *j* is measured using $E_{j}=\ln\left(\frac{1}{\sigma^{2}\left(V_{j}\right)}\right)$ where $\sigma^{2}\left(V_{j}\right)$ is the measure of variance between values in vector *V*_*j*_ which measures the difference in number of times each strategy was used by the participant. Large $\sigma^{2}\left(V_{j}\right)$ value for vector *V*_*j*_ indicates a large difference in the use of different strategies because the participant reported using a subset of strategies more often (i.e., the count values for few of the strategies is large compared to the rest of the strategies). Small $\sigma^{2}\left(V_{j}\right)$ value indicates a small difference in the use of different strategies because a participant reported using a variety of different strategies across the different attempts (i.e., the count values are equally distributed across the strategies). In our data, minimum $\sigma^{2}\left(V_{j}\right)=0.53$ which indicates an almost zero difference in the number of times the different strategies were used by that participant; maximum $\sigma^{2}\left(V_{j}\right)=14.6$.

The measure of exploration *E*_*j*_ is the inverse of $\sigma^{2}\left(V_{j}\right)$ (ln is a natural logarithmic transformation for normalization purpose) and therefore, high *E*_*j*_ indicates high exploration: a participant explored different strategies; while low *E*_*j*_ indicates low exploration: a participant reported to use a subset of different strategies more consistently. We observed a large variation between participants in the extent of their exploration; warranting the test of it's effect on the two outcome variables.

### 3.3. Phishing effort and persuasion performance

*Phishing effort* was measured by the total number of edits (in terms of characters) aggregated over the 8 trials. The number of edits in each trial was calculated using the Levenshtein edit distance function which calculates the number of characters that need to be inserted, deleted and substituted to derive one email from another; objectively quantifying the effort participants exerted in each trial in modifying the phishing email. We observed large differences between participants (μ = 1691.5, σ = 985, *min* = 27, *max* = 4367).

**Figure 6:**
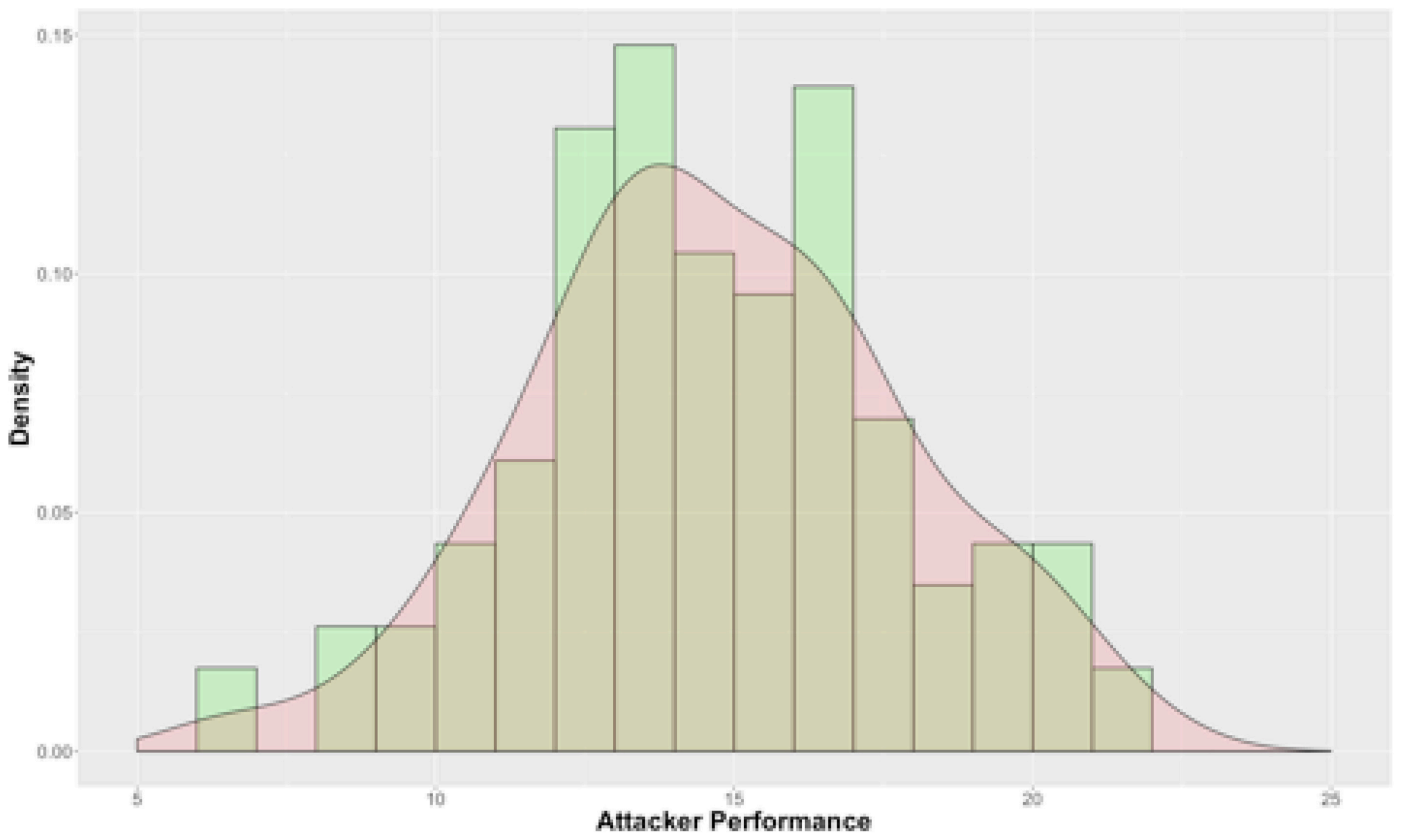
Distribution of aggregated attacker-level performance.

To determine *persuasion performance*, we first aggregated the five different end-user responses collected for each “qualified” (50 edits or greater) phishing email from phase-2 and to create an aggregate email classification score, which ranged from 5 (all 5 end-users chose to delete the email) to 25 (all 5 end-users chose to immediately respond to the email). We then averaged the aggregated email classification scores across all the qualified emails produced by each participant in the attacker role. The resulting persuasion score was normalized to take into consideration the varying number of qualified phishing emails produced by each participant in the attacker role. Figure 6 shows the distribution of persuasion performance. Lower persuasion performance means that end-users deleted the phishing emails whereas higher performance means that the end-users chose to respond to the phishing emails launched. From the Figure 6, it can be observed that a large number of participants scored between 10 and 20 which indicate that while some of their emails were successful in persuading the end-user, others were less persuasive; suggesting the important role of strategies in predicting phishing performance, beyond effect of individual differences.

There was no correlation between phishing effort and persuasion performance (i.e., no relationship between number of edits made to the email and the end-user's response to the email (ρ = −0.14). Hence, the two outcome performance variables are compared and predicted independently.

### 3.4. Phishing effort

A two-way factorial ANOVA was conducted on phishing effort across the two experimental variables: *Phishing Example* and *High-Value Reward Trial*. Result from the ANOVA is presented in Table 2. We found a statistically significant difference in effort according to the trial in which participants received high-value reward. The initial phishing example did not result in a difference in phishing effort.

**Table 2:** Results from factorial ANOVA on total effort.

	***Df***	**Sum Sq**	**Mean Sq**	***F* value**	**Pr (>*F*)**
High-value reward trial	8	15775001.28	1971875.16	2.38	0.0325^*^
Phishing example	9	8658667.26	962074.14	1.16	0.3436
High-value reward trial:phishing example	42	38847243.57	924934.37	1.12	0.3607
Residuals	42	34774959.30	827975.22		

* *p-value < 0.05*.

**Figure 7:**
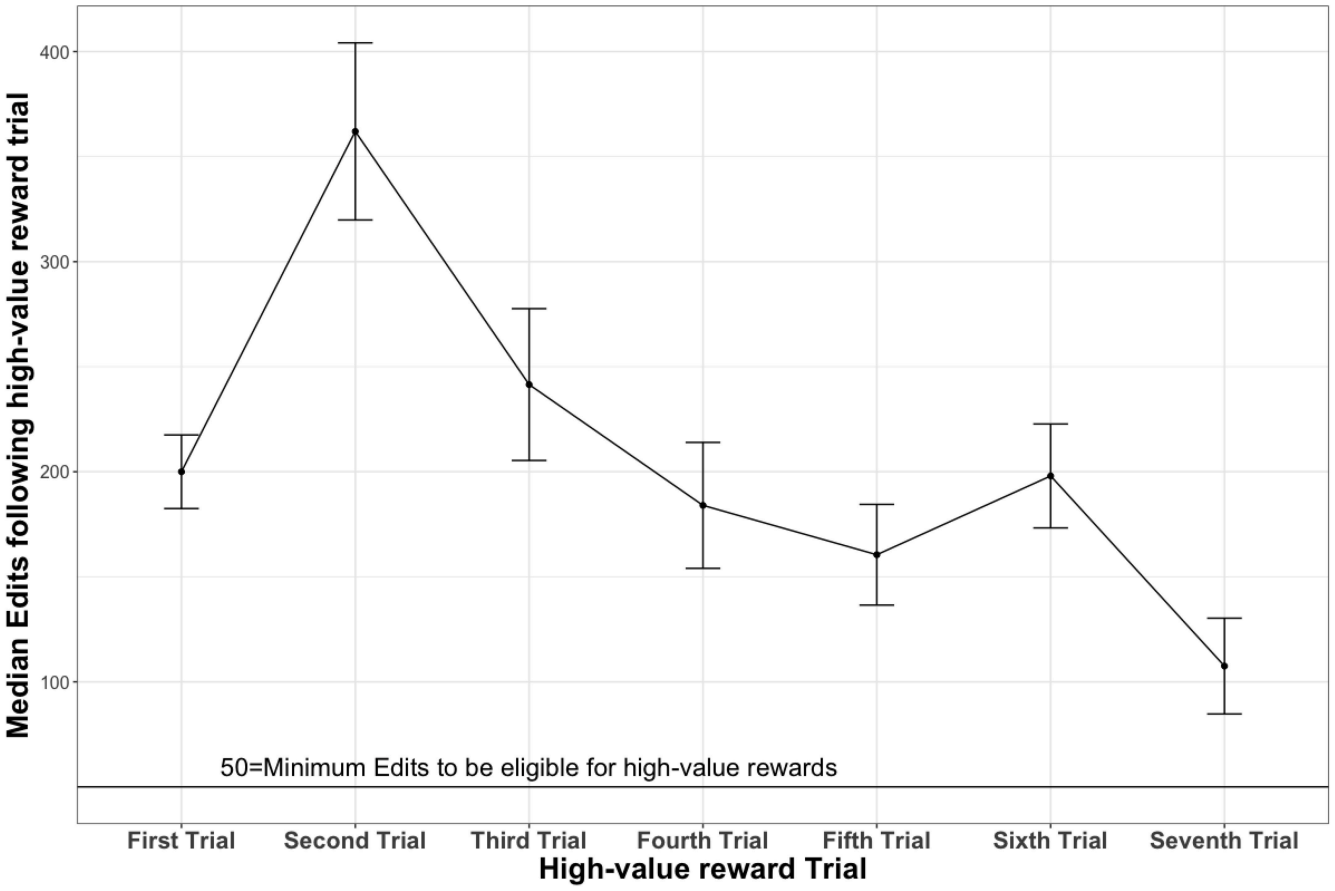
Comparison of average number of edits made in subsequent trials following a high-payoff.

Figure 7 presents the average number of edits made to the emails in trials that followed the trial of the high-value reward. As it can be seen in Figure 7, participants who received the high reward early (e.g., after trial 2) exerted significantly more effort in writing the subsequent phishing emails compared to participants who received the high reward in other trials. Furthermore, the graph shows a decreasing trend in the effort exerted to make more changes in the email as the high-value reward is delayed in the late trials.

A multiple regression analysis was conducted to predict phishing effort in terms of the three behavioral measures: fluency, elaboration, and strategy exploration. The participants' divergent thinking was analyzed using the total number of creative uses reported per participant (i.e., fluency) (μ = 8, σ = 3.3, *min* = 1, *max* = 18); and the average number of words used by participants per use to describe the alternative uses (i.e., elaboration) (μ = 2.4, σ = 1.49, *min* = 1, *max* = 9.7). There was no correlation (ρ = 0.09) between measure of fluency and measure of elaboration which implies lack of relationship between total number of reported uses and the level of details reported per use.

Results from the regression analysis are presented in the Table 3. We found that both fluency and elaboration were significant predictors of phishing effort which indicates that participants who scored high on divergent thinking ability are more likely to put more effort in changing the phishing emails whereas the extent with which they explored different strategies did not have any relationship with phishing effort. The two creativity measures also explained a significant proportion of variance in phishing effort, $R^{2}=0.17,F_{\left(3,\;95\right)}=6.538,p<0.001.$

**Table 3:** Results from multiple regression analysis to predict total effort.

	**Estimate**	**Std. Error**	***t* value**	**Pr (>|*t*|)**
(Intercept)	0.0000	0.0929	0.00	1.0000
Fluency	0.2105	0.0941	2.24	0.0276^*^
Elaboration	0.3606	0.0943	3.82	0.0002^*^
Exploration	0.1619	0.0940	1.72	0.0883

* *p-value < 0.05*.

### 3.5. Persuasion performance

A two-way factorial ANOVA was conducted on persuasion performance across the two experimental variables: *Phishing Example* and *High-Value Reward Trial*. Results from the ANOVA is presented in Table 4. In persuading the end-user to respond, there was a statistically significant difference according to the phishing example email received in their first trial. However, there was no difference in performance between participants who received high-value reward in different trials.

**Table 4:** Results from factorial ANOVA on persuasion performance.

	***Df***	**Sum Sq**	**Mean Sq**	***F* value**	**Pr (>*F*)**
High-value reward trial	8	9.42	1.18	1.45	0.2047
Phishing example	9	22.09	2.45	3.03	0.0073^*^
High-value reward trial:phishing example	40	33.26	0.83	1.03	0.4676
Residuals	41	33.24	0.81		

* *p-value < 0.05*.

Another multiple regression analysis was conducted to predict persuasion performance using fluency, elaboration and strategy exploration as predictors. Results from this analysis are presented in Table 5. We found that strategy exploration was a significant predictor of persuasion performance but in the negative trend which means that participants who explored more strategies across the 8 trials were less successful in persuading end-users. In contrast, participants who explored less strategies were more likely to be successful in getting a response from end-users. We found no evidence of relationship between measures of divergent thinking and persuasion performance. The strategy exploration measure explained a significant proportion of variance in persuasion performance, $R^{2}=0.11,F_{\left(3,\;95\right)}=3.6,p=0.017.$

**Table 5:** Results from multiple regression analysis to predict persuasion performance.

	**Estimate**	**Std. Error**	***t* value**	**Pr(>|*t*|)**
(Intercept)	−0.0000	0.0968	−0.00	1.0000
Fluency	0.0161	0.0980	0.16	0.8696
Elaboration	−0.0269	0.0982	−0.27	0.7850
Exploration	−0.3178	0.0979	−3.25	0.0016^*^

* *p-value < 0.05*.

### 3.6. Association between persuasion strategies

**Figure 8:**
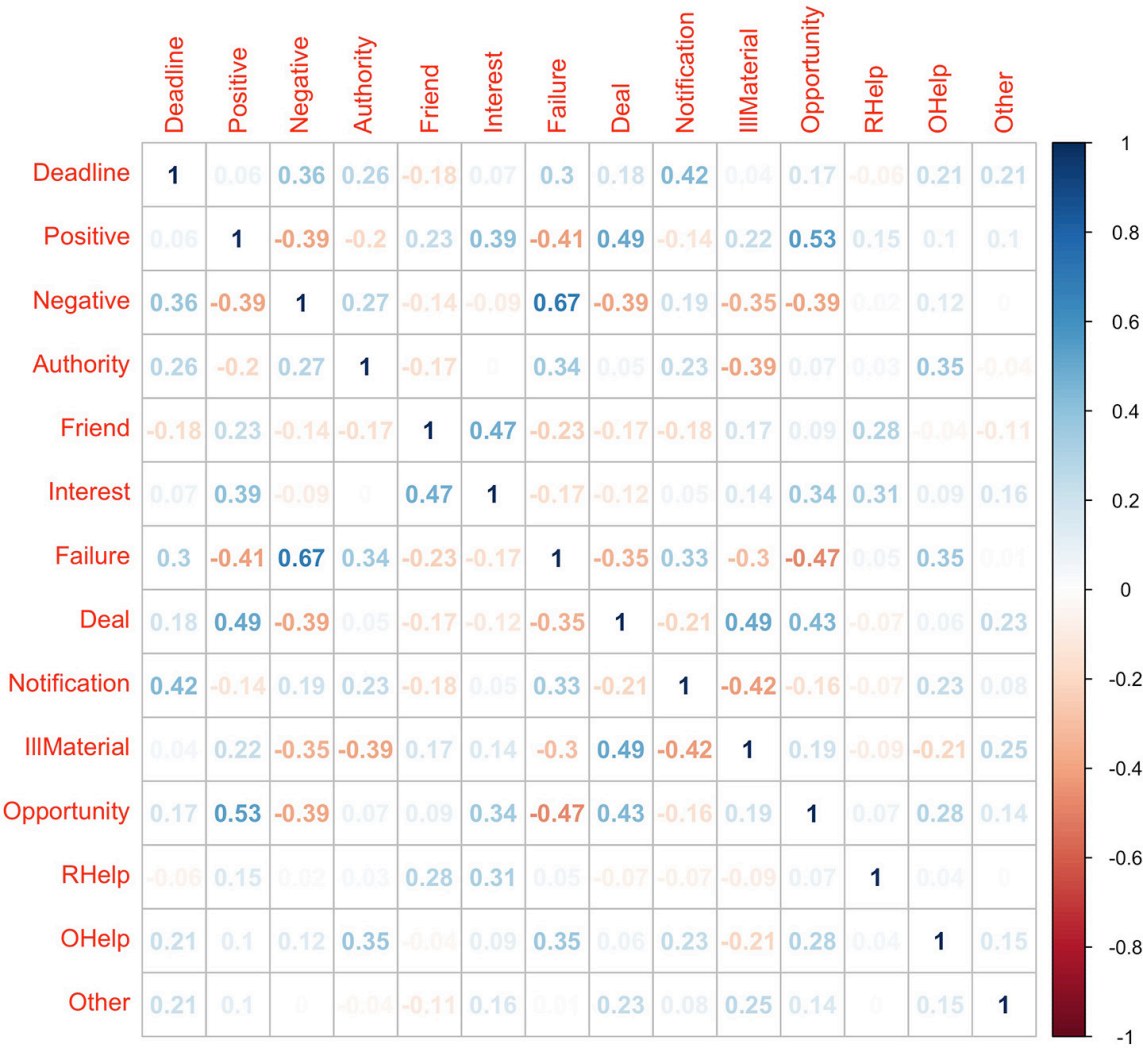
Visualization of pair-wise, polychoric correlation of occurrence between 14 strategies.

Studying the association and correlations between the different persuasion strategies was necessary to understand what strategies were more often used together. Since the strategies chosen for each email are categorical (present or absent), we could not apply traditional correlation analysis. Hence, to analyze correlation between categorical variables, we summarized the frequency of occurrence between every pair of strategies using a contingency table and calculated the polychoric correlation coefficient between every pair of strategies using the corresponding contingency table. Figure 8 shows results from pairwise polychoric correlation between all 14 persuasion strategies.

From the Figure 8, it can be observed that when emails were written with a negative tone, it most often accompanied statements that inform problem/failure/loss and would have contained a deadline as observed with the strong positive correlation between negative and failure strategy (0.67) and with a moderate correlation between negative and deadline strategy (0.36) respectively. In contrast, when emails were written with a positive tone, it most often accompanied statements that offer deal/lottery/reward or presented new opportunity (job, product or service) as observed with the correlation between positive and deal strategy (0.49) and with strong correlation between positive and opportunity strategy (0.53) respectively. Emails that pretend to provide reminder/update/notification are more likely to contain a deadline and may contain statements that inform problem/failure/loss; correlation between Notification and Deadline (0.42) and moderate correlation between Notification and Failure strategies (0.33) respectively. Finally, when emails contain statements that pretend to be from a friend/colleague/acquaintance/relative, it was often associated with statements that pretend to have shared interest (work or activity) as observed with a moderate correlation between Friend and Interest Strategies (0.47).

We also analyzed the relationship between persuasion strategies used in each phishing email and end-user response to each. Since each email was evaluated by five different end-users in phase-2, a mixed-effects regression analysis was conducted to predict end-user responses for each email using the different strategies as predictors (main-effects); end-users were considered the random-effects in the model -using random intercepts per participant- to account for the variance introduced from unobserved participant-specific factors. We also observed that some of the strategies were more often used with multiple other strategies (see Figure 8). So, we excluded strategies “opportunity” and “negative-tone” from the analysis model because these strategies specifically had strong correlations with multiple other predictors (see Figure 8). We also excluded “other” strategy because it was a catch-all, non-interpretable strategy option. Hence, only 11 strategies were used as predictors in the analysis. Results from the analysis is presented in Table 6. Predictors in the model explained a significant proportion of variance in persuasion performance, conditional *R*^2^ = 0.4.

**Table 6:** Beta estimates for 11 strategies from mixed-effects regression analysis to predict aggregated phishing outcome.

	**Estimate**	**Std. Error**	***t* value**	**Pr (>|*t*|)**
Offer a deal	−1.8	0.4	−4.53	<0.05^*^
Sell illegal material	−3.02	1.13	−2.66	<0.05^*^
Use positive-tone	−1.02	0.39	−2.64	<0.05^*^
Use deadline	−0.27	0.37	−0.6	>0.1
Offer help	0.02	0.35	0.06	>0.1
Request help	0.35	0.41	0.82	>0.1
Sound like an authority	0.71	0.39	1.8	<0.05^*^
Send notification	0.82	0.37	2.2	<0.05^*^
Sound like a friend	0.9	0.43	2.1	<0.05^*^
Express shared interest	1.02	0.46	2.2	<0.05^*^
Communicate failure	1.05	0.38	2.8	<0.05^*^

* *p-value < 0.05*.

## 4. Discussion

In contrast to past behavioral studies on phishing, this research highlights results on phishing attacker's behavior. Using a novel two-phase experimental paradigm, we tested the effect of creativity on phishing attack success; measured attackers' exploration behavior with phishing strategies; and compared and predicted phishing effort and persuasion performance.

Regarding phishing effort (measured by aggregating the total number of edits made in emails), we found that effort exerted in a phishing campaign is related to the timing of rewards. The trial in which attackers received a one-time high reward had a significant effect on how much effort individuals applied in writing phishing emails in their subsequent attempts. Compared to participants who received no rewards or who received rewards at the latter stages, those who received high rewards early-on exerted more effort while writing the subsequent phishing emails (Research Question 1). Delaying high rewards resulted in lesser effort (see Figure 7). This result clarifies the effect of delay of rewards in discouraging dishonest behaviors (Wu et al., 2017). The easier and sooner attackers gain high rewards, the more motivated they would become to apply more effort in designing persuasive phishing emails. An implication of this finding is that we need to improve current security practices to change the incentive structure for the attacker (Abbasi et al., 2016). If rewards from attacks are greater than the costs, attackers will continue to exert more effort in their phishing campaigns. We need to determine policies that make it harder for attackers to launch successful attacks (Grossklags et al., 2008; Moore, 2010; Shetty et al., 2010; Fielder et al., 2016). Technological innovations alone are often insufficient to solve this problem. We need to advance phishing training, and security education for general public, enabling them to detect majority of phishing attacks, even the targeted forms of attacks; making it difficult for attackers to gain immediate rewards from phishing emails. To achieve this, we need a better understanding of adversarial behaviors and strategies. In this paper, we contribute a better understanding of how attacker's creativity, their exploration behavior with strategies, and their effort in designing phishing emails may persuade end-users to respond.

Creativity was investigated using divergent thinking measures, particularly fluency and elaboration (Runco and Acar, 2012). We found that participants who scored high in creativity (i.e., reported higher number of alternative uses for “plank of wood,” and described each use with more details) were more likely to spend more effort in developing their phishing emails. We also found that participants who were predisposed to describe the uses with elaborate details were more likely to put more effort, compared to participants who were simply fluent in reporting more number of uses. However, contrary to expectations from the cybersecurity criminal literature (Nikitina, 2012; Steinmetz, 2015), we did not find any evidence for creativity being a key predictor of phishing success (see Research Question 2). Similarly, we also did not find any relationship between individual measures of creativity and the participants' exploration of different kinds of strategies. Hence, we could theorize that attackers with higher creativity could be capable of changing and adapting their emails to evade detection but their creativity may not determine their success in persuading end-users to respond to their emails (Amabile et al., 1994; Mumford et al., 1994; Lakhani and Wolf, 2005).

It appears that perseverance in the use of certain strategies may be a key to success. We found that participants who were more consistent in their use of a subset of strategies across multiple attempts, were more likely to be successful in persuading end-users to respond to their emails (see Research Question 3). In contrast, participants who chose to explore different strategies across their multiple attempts were less successful. It is possible that too much exploration with different kinds strategies could be inhibiting individual's ability to repeatedly improve the email text such that it reflects the strategy effectively. Dilemma whether to explore or persist with a specific strategy for higher productivity has been a long standing question in the management sciences (for example, see Almahendra and Ambos, 2015). A recent fMRI study on exploration and exploitation during individual decision-making, show that exploitation activates regions associated with anticipation of rewards and, regions associated with bottom-up learning processes (learning by doing); whereas exploration is associated with top down learning (driven by experience and knowledge) (Laureiro-Martínez et al., 2015). In the phishing context, we could reasonably presume that majority of attackers have to rely on bottom-up learning process to develop successful phishing emails. This further clarifies our result and inference about the necessity for persisting with a specific strategy to develop successful phishing emails.

It is also possible that certain strategies are inherently more effective than others in persuading end-users to respond. Table 7 presents sections of example emails produced by participants, that represent both successful and unsuccessful strategies. The most successful strategies that were more likely to be viewed and responded immediately by end-users include: (1) send notifications; (2) use an authoritative tone; (3) pretend to be a friend; (4) express shared interest; and (5) communicate failure. In contrast, the least successful strategies that resulted in higher likelihood of deleting the emails include: (1) offering deals; (2) selling illegal materials; and (3) using positive tone. Other strategies such as deadlines, request or offer to help were not found to be predictive of the effectiveness of the phishing emails. This empirical results complements and further clarifies the role of individual strategy on phishing performance (Ferreira and Lenzini, 2015; Ferreira et al., 2015; Zielinska et al., 2016). These results on relationship between individual strategies and phishing outcome answers the research question 3.

**Table 7:** Excerpt of example emails for both ^*^successful and †unsuccessful strategies.

**Strategy**	**Sample phishing email for the strategy**
Offer a deal†	*Records show that you entered to win the state's powerball jackpot …Collect your earnings …Sincerely, Powerball Team*
Sell illegal material†	*Good News! You have been pre-approved for this world class prescription site. …This is a limited time offer so apply quickly*
Sound like a friend^*^	*Are you at your desk? I need you to send me an email attachment with the individual 2015 W-2 (PDF) and earnings summary of all the employees Thank You Sent from my iPhone*
Sound authoritative^*^	*Dear tax payer Our tax records indicate you have taxes owed for the year 2009. …Click here for payment options Sincerely Internal revenue serviceKanasas city MO*
Shared interest^*^	*Hello and good evening. We have just finished reviewing your job application and resume. …Please visit the site below to register and get started*

The example phishing emails the attackers use to motivate their attacks may also play a role in the choice of strategies and phishing success. We found that participants who received an example email in their first trial that conveyed “change of password due to problems in the account” or email that offered deals, were less likely to be successful. In contrast, participants who received phishing examples that contained work-related and social communications, were more successful with their attacks (see Table 4). We provide the ten phishing examples used in this experiment in the Supplementary Material. We however did not find evidence for the effect of phishing example on strategy exploration behavior or effort exerted in creating phishing emails.

Hence, attackers who discover effective strategies and who are persistent in their attempts to making them work would be more successful in persuading end-users to respond to their phishing emails. Our results also suggests an effect of phishing inspiration on phishing success.

The success of these strategies may be explained by how they evoke different behavioral responses from end-users. For example, compared to other strategies, notification of failure would more likely persuade people to respond because of the well-known phenomenon of loss aversion (Kahneman and Tversky, 1979). People may be more averse to accept failure and more willing to take actions on emails that involve possible losses. Phishing emails that use friendly- and authoritative-tone, may evoke peoples' inherent tendency to trust emails with such rhetoric (Cialdini, 2004). Emails that involve unsolicited deals and sale of illegal materials may be ineffective given the familiarity of participants to these type of emails. Currently, people may be less receptive to strategies known to be associated with scams which were effective a decade ago. Incorporating these findings in training programs, is expected to help end-users to detect other phishing strategies that are less common.

In summary, in this paper, we investigated attacker behavior in the phishing context. We analyzed phishing effort and effectiveness. We find that phishing effort is largely determined by individual creativity of the attacker as well as by the incentive structure, where early and high rewards increase the immediate effort that attackers put into constructing phishing emails. We also found that perseverance in the use of effective strategies are key to the success of phishing campaigns. Effective phishing strategies include: sending notifications, use of authoritative tone, taking advantage of trust by impersonating a friend or expressing shared interest, and communicating failure. This work provides insights on the effects of creativity, exploration behavior and strategy choices on the performance of phishing attacks to persuade end-users to respond. These insights may be used for the design of training programs or to improve current anti-phishing technology.

### 4.1. Limitations and future work

Future work could leverage data from this paradigm (phishing emails and strategies) to develop linguistic models that detect adversarial phishing strategies. This paradigm could be adapted to “crowdsource” large number of diverse phishing “intent” email data for training data analytic models. Future work could also test this paradigm as a training intervention for end-users to better detect phishing emails; similar to white-hat hackers, end-users could learn to think like hackers to better detect phishing emails.

In this study, participants were told their goal was to persuade fictional end-users to respond but were not provided any specific details about the targets. This paradigm is however conducive for studies on human behaviors in spear-phishing which has not been previously explored or understood extensively. Future work could adapt this paradigm to study human behaviors in the context of spear-phishing attacks using targeted profiles of victims for eliciting targeted emails and strategies employed in it.

Future work needs to collect data from a more diverse participant sample. For example, in the current sample all the participants in the adversarial role self-reported they were native English speakers, and majority rated their English proficiency as “advanced” or “very advanced”. To study the effect of cultural and language differences on strategies used to build phishing emails it would be important to collect data from a diverse population sample. We did not collect other participant information such as individual experience with Internet/computers/writing emails, but we assume it would be moderate to high among participants from MTurk who use computers, Internet, and emails as primary work tools (Rajivan et al., 2017).

Alternative uses collected as part of the divergent thinking test are scored qualitatively for originality (by comparing uses reported by other participants), fluency (total number of uses), flexibility (different categories) and elaboration (amount of detail) (Runco and Acar, 2012). In this paper, we used only measures for *fluency* and *elaboration* as proxy measures of divergent thinking because an in-depth, qualitative analysis of all the responses to divergent thinking test was beyond the scope of this paper. A comprehensive treatment on the effect of creativity on adversarial performance in phishing attacks should be part of future work.

Access to adversaries is severely limited. Hence, we designed the novel two-phase paradigm and simulation environment presented in the study, where separate groups of participants from MTurk produced (adversary) and examined (end-user) phishing emails respectively. Future work is necessary to compare, quantitatively, the similarity between emails produced from such a simulated paradigm with real-world phishing data sets. Qualitative analysis of the emails reveal that participants from MTurk may be more sophisticated in writing phishing emails than many of real-world attackers. Resulting phishing emails from such a paradigm may also be based on individual's past experience with phishing emails which was not measured in this study. Hence, future work also needs to study how experience affect decisions on the use of adversarial strategies while crafting phishing emails.

## Ethics statement

This study was carried out in accordance with the guidelines of the Office of Human Research Protection (OHRP) and other federal regulatory agencies with written informed consent from all subjects. All subjects gave informed consent in accordance with the Declaration of Helsinki. The protocol was approved by the office of research integrity and compliance at Carnegie Mellon University.

## Author contributions

PR: Generated the initial ideas for the study, designed the experiment, developed the simulation, conducted the data analysis and led the writing of this manuscript. CG: Contributed to crafting the general idea of the research, provided input to make the ideas of this study concrete, helped with the specific decisions on the experimental design, the paradigm and the experimental protocol, and contributed to writing up this manuscript.

### Conflict of interest statement

The authors declare that the research was conducted in the absence of any commercial or financial relationships that could be construed as a potential conflict of interest.
