# Supplementary material for: Creative Persuasion: A Study on Adversarial Behaviors and Strategies in Phishing Attacks
Source: Front Psychol. 2018 Feb 21;9:135. doi: 10.3389/fpsyg.2018.00135 (PMC5826381; doi:10.3389/fpsyg.2018.00135)
Supplement: Supplementary file 1 [file DataSheet1.PDF]

Supplementary Material: Creative Persuasion: A study on adversarial behaviors and strategies in phishing attacks

1 SUPPLEMENTARY TABLES AND FIGURES

Trial	Frequency
0	12
1	11
2	6
3	8
4	8
5	8
6	21
7	21
8	7

Table S1. Frequency of High-value reward across the 8 trials. 0 represents participants who did not receive the high-value rewards.

2 SUPPLEMENTARY MATERIALS

2.1 Phishing Examples Used

Your Account has limitation! You can resolve this now.

Case id : 9000321-128. Login attempt from unknown device.

Dear Client

It looks like someone else may have access to your account, so we have temporarily locked it to keep your personal informations in safe. To unlock your account, you may need to pass a security check. Note that attempting to access someone else is a violation of PayPals terms. It may also be illegal. To reset your account:

1-Click on the link PayPal-Security.html.

2-Open the page in a browser window secure.

3-Follow the instructions.

Account suspended !

108-4596473-8009841 FF

Hello,

We were unable to validate important details about your Amazon Web Services (AWS) Account. Your AWS account has been suspended.

Please visit your account details to confirm the payment information for your account.

Update Your Payment Method

Account Details

Account #108-4596473-8009841

Amazon IT Team

Conditions of Use Privacy Notice 1996-2015

Dear Customer:

We are committed to providing the tools you need to help monitor your account.

Your account has been recently compromised from a different ip location. You are required to verify your account information to prevent account termination.

Click here: https://chaseonline.chase.com/Logon.aspx

Thank you for being our customer. We look forward to serving all of your financial needs.

Sincerely

*********PLEASE DO NOT RESPOND TO THIS EMAIL *********

We are writing to you because your federal Tax payment (ID: 88380290), recently sent is available for refund.

For your security, new changes on the accounts listed above may be declined. If applicable, you should advise any Additional Card Member(s) on your account that their new changes may also be declined.

For more information, please click here

Your prompt response regarding this matter is appreciated.

Sincerely

Dear Sir or Madam:

I am currently pursuing my Masters' in Accounting at the Kelley School of Business at Indiana University. I am also pursuing my CPA following my completion of my degree. Expertise in Microsoft Access, Excel, Word and Powerpoint, Telnet and Ethernet Configuration.

Here is the link to my CV online

Thanks for your pay attention.

Sincerely look forward to joining your team!

Best Regards

Dear user,
The following evaluations have been assigned to you. Please login to complete these evaluations.
CLICK HERE TO LOGIN USING SECURE ENCRYPTION
NOTE: Your login will time out after 60 minutes. Your responses will be lost if you do not click on the "FINISH" button before 60 minutes lapses. There is no prompt when your 60 minute session has expired. Please save extensive comments periodically and check your time.

Elizabeth Hollinger

ITS Account Evaluations

Connected to Microsoft Exchange

© 2014 Microsoft Corporation. All rights reserved.

Disclaimer: This electronic message may contain information that is Proprietary

---

Dear Sir or Madam:

You have an incoming payment directed to this e-mail address. We are unable to process this payment to your account as your details has not been recently verified. Click here to fix this transaction and view your current balance.

Update Your Account Details here

Thank you for banking with us.

Sincerely

Account No: 108-455294-800125-MN

Dear Walmart User,

We at Walmart Online Services are happy to announce that you have been chosen as the 'User of the month' lottery winner. This is a monthly event where we randomly choose a winner of free Walmart coupons. To access your coupons, please visit the link below.

Walmart Reward Coupons

Walmart Online Services

Hello,

Thank you for shopping with us. You ordered “EFFORTINC Vintage Chandelier Deer Horn”.

We’ll let you know once your item(s) have dispatched.

Order Details:

Order # 002-527657-2343090

Shipping: FREE Two-Day Shipping

Order total: $172.34

If you didn’t authorize the transaction, please click here to receive a full refund.

We hope to see you again soon.

Amazon.com

Dear Loyal Customer:

JP Morgan Chase Bank is offering a brand new 30 Year Fixed Term Jumbo Loan with rates starting from 1.05% to qualified customers looking to purchase a house or to refinance.

To see if you qualify, or to talk to a JP Morgan Chase Bank loan specialist, please click the link below.

https://chaseonline.com/promotional/30YearJumbo/qualification.aspx

We look forward to serving all of your financial needs.

Sincerely
